# 4056 Dementia family caregivers’ mobile app use and intention to adopt mHealth apps

**DOI:** 10.1017/cts.2020.194

**Published:** 2020-07-29

**Authors:** Kyra Jennifer Waligora Mendez, Hae Ra Han

**Affiliations:** 1 Johns Hopkins University School of Medicine; 2 Johns Hopkins University School of Nursing

## Abstract

OBJECTIVES/GOALS: To describe preliminary results of Alzheimer’s and dementia caregivers’ (CGs) mobile app use and intention to adopt mHealth apps for their own chronic condition self-management. To discuss implications for designing and implementing mHealth interventions for CGs. METHODS/STUDY POPULATION: This study aims to recruit 110 racially and ethnically diverse family caregivers (CGs) who have a chronic condition, provide care for persons with Alzheimer’s disease or related dementias, and have access to a mobile device. This is a cross-sectional correlational study collecting data with computer-assisted telephone interviews stored through REDCap. The study survey was created using existing surveys about mobile app use; relevant, well-validated research instruments; and questions from the U.S. Census and other national surveys. CGs are being actively recruited from the Baltimore-Washington metropolitan area using various recruitment strategies that have been effective in prior studies. RESULTS/ANTICIPATED RESULTS: The majority of CGs used websites (86%), mobile devices (68%) or apps (53%) to manage their own health. CGs using health-related apps were tracking their exercise (60%), diet (60%), medical records (50%), and physical health measures (50%). More than 4 out of 5 (82%) predicted they would use mobile apps to self-manage their chronic condition, though only 68% actually planned to use them. 86% of CGs were using mobile apps for non-health related purposes, with the most popular app being weather (90%), followed by social media (74%), music/entertainment (68%), and banking/business apps (63%). CGs used weather and social media apps most often (2 or more times/day) and spent 9 hours/week on apps. DISCUSSION/SIGNIFICANCE OF IMPACT: Websites and mobile apps appear to be feasible modes to deliver health interventions to CGs. Researchers should consider including features of apps most frequently used by CGs, such as the weather, ways to connect with others, and music/entertainment, when delivering mHealth interventions to CGs.

